# Integrating perspectives of transgender and gender-diverse youth, family members, and professionals to support their health and wellbeing – a mixed-method study protocol

**DOI:** 10.1186/s13690-024-01270-z

**Published:** 2024-03-18

**Authors:** Julie Servais, Bram Vanhoutte, Sara Aguirre-Sánchez-Beato, Isabelle Aujoulat, Cynthia Kraus, Guy T’Sjoen, Sandra Tricas-Sauras, Isabelle Godin

**Affiliations:** 1 School of Public Health, Université Libre de Bruxelles, CP 596, Route de Lennik, 808, Brussels, 1070 Belgium; 2 Faculty of Psychology and Education, Université Libre de Bruxelles, CP 122, Avenue F.D. Roosevelt, 50, Brussels, 1050 Belgium; 3 Health and Society Research Institute – UCLouvain, Clos Chapelle-Aux-Champs 30/B1.30.15, Woluwe-Saint-Lambert, 1200 Belgium; 4 Faculty of Social and Political Sciences – UNIL, CH-1015 Lausanne, Switzerland; 5 Faculty of Medicine and Health Sciences, Ghent University Hospital, C. Heymanslaan 10, Ghent, 9000 Belgium

**Keywords:** Mixed-methods, Transgender, Gender-diverse, Youth, Co-construction

## Abstract

**Background:**

The current literature highlights a strong link between the poor health outcomes of transgender and gender diverse (TGD) individuals and their negative experiences in various areas of life. Most of these publications rely on adults’ memories, lacking a focus on the current experiences and needs of young transgender and gender-diverse individuals. Furthermore, previous studies on support for these young people often solely consider the perspectives of TGD adults or professionals and rarely involve parents’ viewpoints.

**Methods:**

This study will use a mixed sequential method with a participatory approach. Firstly, the qualitative phase will explore the difficulties and needs of TGD (15–20 years old) and of the families and professionals who support them. Results from this part will be used to develop the questionnaire for the quantitative phase, with the help of a community board. Secondly, based on participatory epidemiological research, the quantitative phase will use an intersectional perspective to measure the impact of individual and structural factors on the quality of life and well-being of transgender and gender-diverse young people. Finally, a co-creation phase will be undertaken to formulate recommendations based on the results of the first two phases.

**Discussion:**

This research aims at better understanding the influence of gender identity on the quality of life and health of TGD young people and their families and to identify protective and risk factors that affect their vulnerabilities.

**Ethics and dissemination:**

This study has been approved by the Ethics Committee of the Erasme Faculty Hospital (CCB B4062023000140). As this research is participatory and part of a PhD dissertation, we aim to disseminate the results through our partners’ networks and structures locally, and internationally through conferences and peer-reviewed journals.

## Background

The lack of inclusion of lesbian, gay, bisexual, transgender, queer, intersex, asexual (LGBTQIA +) communities is a significant issue in schools, sports, work, and healthcare [1–5]. Discrimination, integration issues, greater risk of harassment are all challenges faced by young LGBTQIA + people [6–8]. Previous research revealed the many aspects of their oppression, all of which have deleterious effects on the health and well-being of this population [9–12]. Similarly, limited access to health care and quality care [13, 14], substance use [15], greater risk of harassment [11, 12], homelessness and poverty [16–18] all contribute to the potentially poor health of transgender and gender diverse (TGD) people.

The period between 15 and 20 years of age is widely recognised as a period of life characterised by both physical and psychological profound changes as well as changes in the social and family sphere [19–21]. It is a period marked by the transition from a vertical socialisation provided by the family home to a horizontal socialisation characterised by a detachment from the home and increasing closeness to peers [22, 23]. During this period, young people may be confronted with specific vulnerabilities that can endanger their future prospects such as discontent, self-esteem and self-image disorders, family breakdowns, etc. [21, 24, 25]. These vulnerabilities can be amplified in a situation of gender non-conformity because the youth has to deal with gendered aspects of bodily changes, identity issues, the gaze and opinions of his or her family and the judgement of others [26, 27]. Although there are few qualitative studies on the experiences of young TGD people [28], the frequency of negative experiences related to the gaze or opinions of others seems to be linked to the age of the young person at the time of the occurrence. They are more prevalent in primary and secondary schools than in tertiary education, according to the testimony of TGD adults [29].

Gender non-conforming can cause difficulties in family life, social life, school life and other important areas, especially in the 15–20 age group [15, 30, 31]. Yet parent- or youth-initiated counselling often lags, partly because the topic is still taboo in many families, regions and countries [32, 33]. The literature indicates that most parents accept that there is variation in their child’s gender expression on a transitory basis [34–36]. However, when this persists, they become concerned about their child’s psychosocial well-being. Unsure of the appropriate way to deal with the issue, most of them seek help and support by taking the step to consult a mental health professional together [34–38]. In other cases, parents find it more difficult to accept a young person that does not correspond to their gender expectations, notably because of cultural or community pressures and/or their own beliefs [39].

Regarding *‘generic’* health care, several studies have shown that the main barriers to quality care are related to the lack of training of health care professionals as well as to their representations regarding the TGD youth [40, 41]. Indeed, family doctors and psychologists that are not specifically active or trained in the field of TGD care seem to lack the relevant and useful information to meet the needs of this population and to appropriately refer them and/or answer their questions [29, 42–45].

To identify the age-specific needs of young people, awareness raising in schools is necessary [46–49]. Educational professionals can also play a key role in the acceptance and affirmation of a young person’s gender identity by having a supportive and caring attitude [29, 49, 50]. But when faced with difficult situations at school, such as access to locker rooms or gymnastics classes that are still too cisnormative, or when subjected to bullying based on gender identity, professionals such as psycho-medical-social workers generally don’t have much knowledge or answers to offer on TGD-related issues [49, 50].

While TGD people are becoming more visible, with media coverage of some artists coming out and several films or series following the journeys of TGD people, and while Belgian policy emphasises the promotion of sexual, emotional and relational health (especially in schools) to reduce gender-related social and health inequalities, current literature shows that negative health outcomes for TGD people are still strongly correlated with negative experiences in their own environment, such as—family, school, health care, etc. [15, 49, 51–53]. However, these findings are often the result of retrospective accounts from adults and there are very few studies on the current experiences and needs of TGD youth. According to Költö, this is one of the current research gaps, at least in Europe, for this community [28]. Furthermore, previous studies on the issue of support for young TGD people have approached it either from the perspective of TGD people or from the perspective of professionals, but rarely from a combination of the two and fail to consider parents’ perceptions. The aim of the project is to co-construct recommendations and proposals for relevant support strategies in order to improve the well-being of TGD young people in their different life settings.

### Theorical underpinnings

This research project will use the framework of the bio-ecological model [54–56] and intersectionality theory [57].

Bronfenbrenner’s bio-ecological systems theory was developed to understand human development within various interconnected systems [54–56]. The subsystems identified by Bronfenbrenner include: 1/ the microsystem (directly interacting groups such homes, schools, or religious communities); 2/ the mesosystem (relationships between two or more microsystems, such as school and parents); 3/ the exosystem (environments influencing development without direct influence, such as the media); and 4/ the macrosystem (broader systems encompassing community, culture, and politics) [54–56]. Finally, chronosystem includes the experiences and life changes of youth over time, both personally and socio-culturally, as well as their individual developmental trajectories. This theory has been applied to a variety of contexts, including to transidentity [58–60].

While recognising individuals are situated at the intersection of various systems, ecological system theory places less emphasis on how social group membership impacts their experiences within these contexts. This is exactly what intersectionality theory emphasizes: individuals’ experiences and functioning are strongly influenced by the interplay between social categories (e.g., ethnicity, social class, gender, sexual orientation) in multiple systems of oppression and privilege [57, 61].

Nevertheless, while intersectionality highlights the multiple and interconnecting systems that perpetuate inequality and opportunity [57], it lacks an explicitly developmental dimension [62]. By merging intersectional and ecological perspectives, as demonstrated by some authors [62–64], it becomes possible to achieve a comprehensive integration of each identity, their intersections, and their interactions with different subsystems. In essence, this approach aims to establish connections between different systems of oppression (e.g., racism, transphobia, classism) and contexts (e.g., family, school, neighbourhood) that are intricately intertwined.

## Methods

### Study aim

This research project seeks to gain a better understanding of the influence of gender identity on the quality of life and health of TGD adolescents and young adults (AYAs) (15 to 20 years old) and their families, and to identify the risk factors that increase their vulnerability. The ultimate aim of this research will be to co-construct intervention and support approaches based on the preferences and needs of TGD AYAs and their families, as well as on the needs of the people who support them. In this sense, participatory research methods will be favoured as they enable the sources of marginalisation to be identified, understood, and addressed in close collaboration with communities such as young TGD people. Moreover, this participatory approach has the potential to cultivate collaborative relationships among individuals engaged in the lives of these youths with various gender identities. This promotes a collective comprehension of the most efficient methods for shaping systems and instigating profound change [65–67].

This main objective and methodology give rise to three areas of research linked to interdependent secondary objectives:

1. *Qualitative study* to address the difficulties and needs of TGD young people by integrating their perspective, knowledge, and experience as well as those around them namely parents, siblings, extended family, and professionals.
  To understand and characterise the representations of gender diversity, the lived meaning, and perceptions of TGD AYAs currently living in French-speaking Belgium.
  To identify the resources (social, professional and/or other) that young TGD AYAs use and the types of additional support they would need.
  To understand and characterise the representations and perceptions of the experiences of young TGD AYAs through the eyes of parents (and extended family) and professionals who accompany them.
2. *Quantitative study* to be designed on the basis of the results of the qualitative component and designed in collaboration with the expert group. This part aims principally to assess whether there is a difference in quality of life, self-confidence, and satisfaction with the support between the different TGD identities through the lens of intersectionality, and if so, to describe the nature of this difference.
  To describe and clarify the average scores on outcomes listed above, considering gender diversity beyond binarity.
  To describe and clarify the average scores on outcomes above while considering the intersection of different social locations, power relations and experiences.
3. *Co-construction* to produce recommendations on the basis of the results of the qualitative and quantitative data collection. The aim of this section is to help build a more inclusive society that takes into account the diversity of genders and identities, and to improve support for young TGD people by taking into account their perspectives, their needs and those who support them. (parents, extended family, professionals).

### Study design

**Fig. 1:**
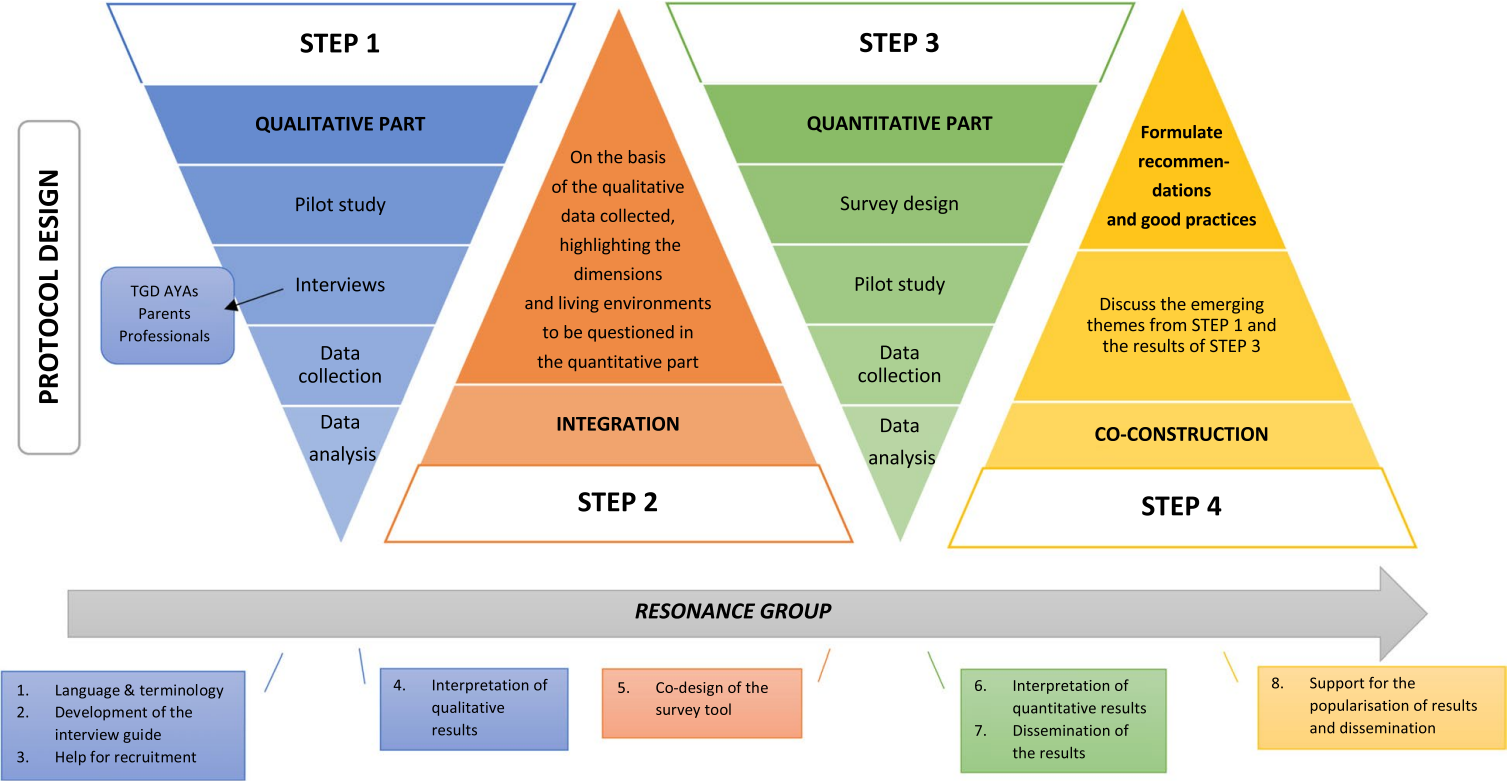
Research design—sequential mixed methods

This project will use mixed methods by conducting both qualitative and quantitative research in a sequential manner (see Fig. 1). As some authors point out, the combination of qualitative and quantitative approaches allows for a greater in-depth understanding of the results for the study of complex social, behavioural and health phenomena [68–70]. An additional reason for using a mixed method approach is the possibility to triangulate the results, which assumes that ‘the use of different sources of information will confirm and improve the clarity of a research result’ and in the case of this project, the relevance of the recommendations [71]. The qualitative study aims to understand the representations, perceptions and lived meanings of gender identity of the different stakeholders. Due to our research priorities, the qualitative component will be strongly developed due to the lack of research in this area in French-speaking Belgium, particularly in our study population. From a sequential exploratory perspective, identifying the living environments that are important for young people with TGD and their needs in terms of guidance and support will enable us to design the questionnaire for the quantitative part of this research [72]. This part will enable us to reach a wider population and see whether the living environments and support needs identified are the same according to the internal diversity of gender identities within the TGD population.

### Resonance group

Co-construction methods allow us to bring together different types of knowledge (experiential, professional) without taking into account a hierarchy between them [73]. Therefore, from the start of the project, our methodology will include the creation of a resonance group composed of TGD adults, family members (experiential knowledge), and field professionals (professional knowledge). Throughout the project and beyond, the members of this resonance group commit to accompanying and questioning the researcher individually and as a group, based on their expertise and availability. This group will guide the researcher through the different parts of the project (see Fig. 1), such as 1/ language and terminology, 2/ development of the interview guide, 3/ recruitment in both qualitative and quantitative parts, 4/ interpretation of qualitative results, 5/ co-designing the survey tool, 6/ interpretation of quantitative results, 7/ support for the dissemination of the results, 8/ participation in the co-construction step and 9/ clarification and reflection on our position throughout the research process.

### Qualitative phase

#### Participants

Participants in the qualitative part of the study will be from three stakeholder groups, transgender youth, parents (and/or extended family) and professionals. The study will include participants from French-speaking Belgium and Brussels.

##### Transgender youth

For the semi-structured interviews with young TGD people, we aim to include French speaking young people aged 15–20. They will be recruited through different networks: 1/ via trans-specific care consultations; 2/ via partner associations in the research project; 3/ via social networks. Depending on the location, professionals will either be asked to inform young people and their parents about the research project, or they will select young people capable of participating in this type of project beforehand. At first, the Schedule for the Evaluation of Individual Quality of Life (SEIQoL) tool will be used to encourage young people to narrate what they consider essential to their quality of life [74]. The semi-structured interview, which is central to the approach, invites the participants to express themselves initially on the dimensions that are most important to them in terms of quality of life, then on their current level of satisfaction in each of the dimensions. Finally, they are asked to rank the dimensions that make up their quality of life according to level of importance [75, 76]. Following this assessment, we will ask the young people to take one or two photos per dimension (those mentioned by the young person), and we will schedule a second interview with them. Photovoice often enables comprehensive data to be obtained by facilitating a relationship between the participant and the interviewer and by encouraging participants to provide an in-depth understanding of their experiences in terms of emotions, feelings and ideas [77]. This method is a form of participatory method and is particularly suited to research with adolescents and young people [78–80].

##### Parents and professionals

Parents (and extended family) and professionals’ recruitment will be undertaken via 1/ trans-specific care consultations; 2/ via partner associations in the research project; 3/ via social networks; 4/ via email or phone call campaigns to professionals working in the areas highlighted by the interviews with young people. Data will be collected through semi-structured interviews supported by a thematic interview guide as the content helps to sustain the discussion but leaves it open to flexibility and creativity [81]. The interview guide will be developed with the resonance group and based on the literature. Given the sensitive nature of the topic, we will consider the use of tools such as vignettes, photos, or any other relevant instrument to facilitate dialogue and reduce participant reluctance [82–84]. Through these interviews we would like to understand how the different stakeholders perceive the current organisation of the comprehensive care system in relation to the needs highlighted by the young people. We would also like to understand how they perceive their own needs and the specific needs of young TGD people are being currently considered and what potential improvements they would like to see introduced into the Belgian system to improve the inclusion of this community.

#### Data collection

With the consent of participants, the sessions will be recorded. This will allow us to fully concentrate on the interview. The recordings will be transcribed *ad verbatim* and analysed thematically, based on the content of the answers, and iteratively. Based on the first interviews, an initial list of emerging codes will be identified and organised in a tree structure. The theme is identified inductively: once identified, this theme is compared with other data to confirm its presence in other interviews (deductive approach) [81, 85, 86]. The data will be compared between the interviews of the TGD young people, the parents, and the professionals to make an initial theoretical comparison. In a later stage, we will bring them into dialogue with theoretical constructs from the literature. Upon completion of the analysis, the recordings will be destroyed.

### Quantitative phase

#### Participants

When collecting qualitative data, we chose not to select our participants based on their gender identity. Nevertheless, several articles highlight the importance of considering the internal diversity of the TGD population in research [15, 52, 87–89]. Therefore, we plan to undertake a quantitative data collection on gender plurality in French-speaking Belgium with an online survey tool.

#### Sample

Survey participants will be recruited using two methods: either in person (through events, community gatherings, trans-specific care clinics) or online (through mailing lists, social networks). The eligibility criteria will be: 1/ to identify as a TGD person; 2/ to be at least 15 years old and less than 20; 3/ to live in Belgium; 4/ to be able to understand French, or English. A convenience sampling approach within the TGD population will be used. Although there is no precise data on the size of the TGD population worldwide and figures depend on the definition of transidentity used, estimates suggest a prevalence of 0.7% to 2.5% among young people aged 15 to 20 [15, 90–93]. In French speaking Belgium, based on the age pyramid, transidentity would therefore concern between 2,472 and 8,827 young people between 15 and 20 years of age [94]. For this reason, it would be ideal to have a sample size of at least 300 people, i.e. an estimate of 5% of the population of young people targeted by the study, in order to have sufficient statistical power to carry out the analyses.

#### Type of study

This is a cross-sectional observational study. Like the other parts of this research project, this study will be based on four of the principles of participatory research as defined by Israel et al. [95] and adapted by Bach et al. [96, 97] to fit participatory epidemiology: 1/ joint definition of objectives and research questions, 2/ joint definition of the populations studied and their health-related contexts, 3/ selection or development of appropriate survey instruments, and 4/ dialogical forms of interpretation of results. We therefore cannot add supplementary data as the questionnaire will be based on findings from the qualitative data and constructed with the help of the resonance group and some of the people interviewed in the first part of the project and who are willing to participate [98, 99]. Nevertheless, some parts of the *Health Behaviour in School-aged Children (HBSC)* study will be used as a baseline. HBSC is an international cross-sectional school survey conducted every four years in around fifty countries [100], including Belgium. Data on health status, health behaviours and well-being are collected from children and adolescents between 10 and 21 years of age.

#### Data analysis

Data analysis will be undertaken using Stata 17.0 software. All variables included the study questionnaire will be analysed. Missing data will be reported. For the description of the sample, the qualitative variables will be described and compared using the Pearson’s Chi-2 test or Fisher’s exact test if the former is not applicable.

Then, the data analysis will be divided into two stages: bivariate analysis and multivariate analysis. The bivariate analysis will consist of measuring separately the strength of the associations between gender identity and each of the exposure factors, without adjusting for potential confounders. The multivariate analysis will be performed using adjusted multivariate regression models with binary and non-binary identities as the main statistical predictor of social and health outcomes, adjusted for the following control variables: age, education, ethnicity, region of residence [101].

### Co-creation phase

This part of the project will focus on comparing and contrasting the perceptions of young people, parents and professionals regarding gender identity and will allow to co-construct with them recommendations for good practice around non-medical support for young TGD people. We will encourage group discussions with an emphasis on participatory methods to empower the different actors [82, 102–105].

#### Participants

The resonance group and the participants interviewed in the first part of the project will be invited to participate in this stage of the project. Each person interested in participating in this group, whether they were interviewed in the previous phase or came through another channel, will be asked to fill in a description form and explain why they wish to participate in this group. This information will allow for the composition of the groups to be balanced, ensuring a good flow of discussion between participants, as recommended in co-construction methods [73, 103].

To prepare TGD young people to participate in larger groups, we will meet them first through focus groups. In a second phase, more diverse groups will be organised with 8–12 participants, mixing equally TGD youth, parents (and family members) and professionals. They will be invited to regular participation like communities of practice to promote the co-construction of knowledge through an iterative process [103, 106].

#### Course of the activities

The members of each group will be asked to respect the confidentiality of exchanges and any other provision that the group members consider necessary for their proper functioning [107, 108]. Prior to the meetings (4 sessions will be planned), preparatory material will be communicated to the participants, either for individual appropriation or during a collective session between participants. Indeed, the co-construction of knowledge is an iterative process, which proceeds in sequences: ideally, the participants alternate times of individual and collective work [106].

Each participant will also be asked to keep a virtual or paper log of their experience [109, 110]. If participants wish to do so, they may share all or part of this with the researcher to illustrate this in the final manuscript. An external support person will be identified and will participate in the different groups to ensure the psychological safety of the participants: [111].

These group discussions will have two purposes: 1/ to discuss the emerging themes from the interviews and the results of the quantitative data collection; 2/ to formulate recommendations and good practices based on these results.

#### Data management and analysis

Thanks to the recording of the discussions (with the consent of the participants), a report will be written for each group session, summarising the discussions, the elements to be included in the analysis of the results of the interviews and the elements that the participants wish to use in the next session. The data collected will be used to inform the analysis of the interviews and potentially to undertake additional analysis if new data is collected during the discussions. The minutes will be shared with the participants and will only be accessible to members of the working group.

This will therefore be used to support the integrated findings of the qualitative and quantitative parts and to help formulate recommendations.

### Ethical considerations

When considering research, particularly when involving sensitive issues and adolescents and young adults, ethical aspects are essential to consider [112], which is why the research protocol was submitted to the Ethics Committee of the Erasme Faculty Hospital and received its approval on the 27^th^ of July 2023 (CCB B4062023000140).

Before submitting the research protocol to the ethics committee, a literature review was carried out to identify the methodological and ethical challenges associated with participatory research with TGD young people. A secondary objective of this literature review was to highlight the considerations when parental consent is required, as obtaining parental consent may compromise their safety, well-being, or privacy, if they live in case their family is not supportive [113–115]. This issue puts young people’s rights to autonomy, privacy, and freedom in tension with parents’ rights to protect their children and is perhaps one of the reasons why findings about the TGD community are often the product of adult retrospective accounts [112, 114, 116].

For the qualitative study, we will pay particular attention to the participation of minors in this project: consent will be adapted, and they may be accompanied by a trusted person. In addition to a description of the project, a consent form will be provided to each participant in order to collect their consent and to provide them with information on confidentiality, their right to access the data, their right to rectify it if considered to be incorrect, their right to object to its the use, and the right to be forgotten. The participants’ identity will be protected by using pseudonyms, and any data that may identify an individual will not be transcribed or coded. Pseudonyms will be used on all files and transcripts. Each participant will be given a copy of the transcript of their interview on request. All the data used are solely for research purposes in the context of this project. Finally, in. regards to the risks of participating in research in a health field that includes vulnerable populations, we will provide a contact person to liaise with participants [112]. For the quantitative study, we will not include any identification information as it will be an anonymous survey. An information and consent section will be provided before participation in the survey.

### Dissemination

Participative research emphasises on prioritising the experiences, perspectives, and actions of participants (e.g., storytellers, data producers) over academic researchers. The goal is to safeguard participants’ narratives, stories, and their roles as ‘tellers’ or authors from being dismissed or silenced [117]. Therefore, an emphasis on the principles of dissemination as described by Israel [95] will be made. One key principle in this regard indicates that the researcher must consult participants before submitting documents for publication, acknowledge the contributions of participants and, where appropriate, develop collaborative publications [95].

As this research is part of a doctoral thesis, dissemination will also be carried out through its publication as a dissertation, as well as through several scientific articles and presenting of results at conferences. The members of the resonance group will be able to ensure their own dissemination plan with or without the help of the researcher for dissemination of results. Particular attention will be paid to translating research findings into understandable language so that they can be disseminated as widely as possible [118].

## Discussion

This research aims to better understand the influence of gender identity on the quality of life and health of TGD young people and their families and to identify protective and risk factors that decrease or increase their vulnerabilities. However, the approach chosen for this study presents several challenges. Ethically, like many other countries, Belgium requires parental consent prior to participation in medical or non-medical research [119, 120]. This is known as the *statutory approach* to consent, in other words, the legal approach to consent. Although seen as an important safety barrier and good practice, the requirement for parental consent is open to criticism as it may discourage young people, particularly those who are marginalised, from participating in research [112, 115, 121–124]. For this reason, this project will favour a *maturity or skills-based approach*, which values young people’s agency. This approach highlights the fact that young people’s ability to understand is dynamic and developing, and is most certainly influenced by their life experiences and socio-cultural contexts [125].

Beyond the question of consent, and from a methodological perspective, the specific participation of young people in this type of research also highlights some important issues. Firstly, like other social groups, young people are not a homogenous group. Secondly, factors such as socio-economic class, ethnicity, culture, and environment play a very important role in their life experiences. Although participatory research does not necessarily prioritise representativeness, it is often more beneficial to involve some young people while being aware of and acknowledging the voices that are included or overlooked, rather than conducting research without any input from young people. A combination of data collection methods can then enable us to hear the voices of those who were not reached in the qualitative part. In addition, participatory methods pose challenges in terms of negotiating boundaries and power dynamics, especially when involving young people. Firstly, researchers need to move from their traditional role of producing results and recommendations (problem definition) to the role of facilitators, working with communities to find solutions that meet their needs [126]. Secondly, in order to avoid adultism (where young people are marginalised because of their age and experience), they need to re-conceptualise the relationship between adults and young people as an equal one [127].

This research project also has strengths. The creation of a resonance group to overcome the challenges mentioned above is considered to be a strength. Indeed, this group will help us ground our research locally, including recruiting these young people. It will also ensure that the study and its methods are acceptable to the community [105]. The use of mixed methods is also a strong point of this research project, as these methods allow a better understanding of complex social phenomena [68, 70, 72].

At the macro-level, the study may help to address important policy and research questions. The knowledge co-construction approach suits to influence policy by generating evidence and supporting citizen participation—understood as the involvement of actors in society in the broadest sense [128]. Therefore, political authorities are likely to be interested in the results of our project, especially given the development of various policies that consider LGBT + communities.

## Data Availability

No datasets were generated or analysed during the current study.
